# Correction: Investigation of injury severity in urban expressway crashes: A case study from Beijing

**DOI:** 10.1371/journal.pone.0236911

**Published:** 2020-07-23

**Authors:** Quan Yuan, Xuecai Xu, Junwei Zhao, Qiang Zeng

The following information is missing from the Funding statement: This study was supported by the National Key R & D Program of China (2017YFC0803802).
