# Supplementary material for: Investigation of injury severity in urban expressway crashes: A case study from Beijing
Source: PLoS One. 2020 Jan 13;15(1):e0227869. doi: 10.1371/journal.pone.0227869 (PMC6957292; doi:10.1371/journal.pone.0227869)
Supplement: Attachment — Submitted filename: Response to Reviewers Comments.doc [file pone.0227869.s001.doc]

# Responses to Reviewers’ Comments

Dear Editor,

# Thanks for the suggestions of our manuscript entitled with “*Investigation of injury severity in urban expressway crashes: A case study from Beijing*”. The revisions are as follows according to the reviewers’ comments and are highlighted with red color in the paper to reveal the improvement.

We greatly appreciate your time and efforts to accept our manuscript for publication. Please contact me freely if any problems come up.

Best regards

Xuecai Xu

**Reviewer #1**:

This is an interesting study that focused on the crash injury severity. The overall analytical works and results are sounding. The followings are my comments and suggestions.

1. Did the authors also obtaining the Property Damage Only crashes? It seems that only injury and above severity crashes were collected. At least the readers should be informed with this kind of information regarding the quantity and the proportion of the severe crashes.

**Response:** Different agencies may classify the injury severity into different types, e.g. Xu et al. (2019), Xu et al. (2018), and Xu et al. (2016) represent the classification in Nevada, Croatia, and Hong Kong, respectively. In this study, the data were collected from Beijing, China, and the classification is divided into slight, injury and fatality. After we check out the dataset, slight here includes property damage only, which accounts for a small proportion in the slight type. Table 1 gives the proportion of three severity types.

References:

Xuecai Xu*, Dong Huang, Fengjun Guo. Addressing spatial heterogeneity of injury severity using Bayesian multilevel ordered probit model. Research in Transportation Economics, 2019. <https://doi.org/10.1016/j.retrec.2019.100748>.

Xuecai Xu, Željko Šarić*，Feng Zhu, Dario Babić. Accident Severity Levels and Traffic Signs Interactions in State Roads: A Seemingly Unrelated Regression Model in Unbalanced Panel Data Approach. Accident Analysis and Prevention, 120, 122-129, 2018

Xuecai Xu, Siqi Xie*, S.C.Wong, Pengpeng Xu, Helai Huang, Xin Pei. Severity of Pedestrian Injuries due to Traffic Crashes at Signalized Intersections in Hong Kong: a Bayesian spatial logit model. Journal of Advanced Transportation, 50(8), 2015-2028, 2016.

2. For the variables considered, as a recent study claims that speed has great influence on the urban expressway crashes. (Rongjie Yu*, Mohammed Quddus, Xuesong Wang, Kui Yang, 2018. Impact of data aggregation approaches on the relationships between operating speed and traffic safety. Accident Analysis & Prevention 120, 304-310. ) However, from Table 1 it seems the authors did not consider this type of parameter. Please justify.

**Response:** Thanks for the suggestion. Various studies have verified that travel speed is indeed significantly associated with traffic safety. In our study, speed limits around all the expressways have been collected, but travel speed wasn’t obtained due to the limitation of data collection. This point has been explained in the weakness in the last paragraph.

As known to us all, actual travel speed is hard to obtain because not each crash can achieve the travel speed, i.e. some may be the range (higher or lower than certain speed) to determine the relation to the speed limit, and then decide whether it exceeds the speed. Moreover, most of the crash results are determined from the video recorded or experience to estimate, and there exists some uncertainty since not all the vehicles have been installed the black box or travel recorder.

*“Another issue is that travel speed may be significantly associated with traffic safety [22], and although speed limits have been collected in this study, they are not reflected from the actual modeling process.”*

3. Bayesian inference different from the traditional statistical model that they do not have a likelihood function. However, the authors listed both likelihood values for the two types of models. Please justify this issue.

**Response:** Bayesian inference gives the log marginal likelihood, instead of likelihood directly. In order to make the comparison equally, Bayesian multilevel ordered logistic model is presented as shown in Table 2.

**Table** 2 Parameter estimates for the proposed models

| **Variable** | **Bayesian multilevel ordered logistic** | | | | **Bayesian ordered logistic** | | | |
| --- | --- | --- | --- | --- | --- | --- | --- | --- |
|  | Mean | Std. Dev. | MCSE | 95% BCI | Mean | Std. Dev. | MCSE | 95% BCI |
| Crash location | -0.467* | 0,201 | 0.013 | (-0.863,-0.067) | -0.409^*^ | 0.207 | 0.009 | (-0.831,-0.021) |
| Time | 0.896* | 0.365 | 0.026 | (0.205, 1.637) | 1.000^*^ | 0.372 | 0.025 | (0.224,1.724) |
| Crash season | 0.501* | 0.211 | 0.025 | (0.102,0.910) | 0.554^*^ | 0.209 | 0.009 | (0.160,0.964) |
| Cut1 | -2.127 | 0.798 |  |  | -1.785 | 0.773 |  |  |
| Cut2 | 1.184 | 0.765 |  |  | 1.494 | 0.732 |  |  |
| Sigma^2^ | 0.277 | 0.460 |  |  |  |  |  |  |
| **Goodness-of-fit** | | | | | | | | |
| **No. of observations** | | 133 | | | 133 | | | |
| **DIC** | | 223.312 | | | 222.297 | | | |
| **Log marginal likelihood** | | -123.916 | | | -124.731 | | | |

**Reviewer #2**:

In the present study, a Bayesian ordered logistic regression model is proposed to investigate the impact factors for injury severity in urban expressway crashes. The content is well organized. However, I have several concerns as follows.
1. The manuscript employed the ordered logistic model. However, in P2 ln 32, according to the literature review, the authors concluded that “ordered logistic/probit model” is applicable. I wonder why the ordered logistic model is selected?

**Response:** Technically, ordered logistic and probit models have the same modeling process, and there is no big difference. The only difference lies in the model results: the former one gives the number, while the latter shows the probability. Thus, there is no preference for both models, and either is suitable.

2. Please double check the crash counts in Figure 1. The authors claimed that 133 samples were kept, However, there were 134 crashes in the figure (46+36+6+39+7=134). By the way, it would be better to give a brief description about the reason why 33 records were deleted.

**Response:** Thanks for the reviewer’s reminding. The crash counts in Figure 1 have been revised and the samples have been corrected. The reason that 33 records were deleted has been added:

*“Since one crash may involve more than one vehicle, some data were double counted.”*

Figure 1 Study Area by Selected Expressways in Beijing

3. P3 ln 17, it is easy to see the difference between injury crash and fatality crash. But how about the injury severity type of slight. And I wonder is non-injury crashes included in this study. If not, please give an explanation.

**Response:** As explained in the response to Reviewer #1, property damage only is included in slight severity.

4. P5 table 1, the “S.D.” is not provided. And in order to provide a comprehensive data description, I would recommend the authors provide an injury-specified data description.

**Response:** Thanks for the reminding of “S.D.” in Table 1, and the values have been added. The data description has been added before Table 1.

5. P6 ln 27-30, please double check the log marginal likelihood value. In general, higher log likelihood, or say lower absolute value, indicates better goodness-of-fit. And it is really confusing that how can the authors get the log marginal likelihood value for a model estimated using maximum likelihood estimation approach. In addition, there seems no significant difference between the estimated coefficients in the two models.

**Response:** In order to make the comparison equally, Bayesian multilevel ordered logistic model is presented. The revision is as follows:

*“…multilevel ordered logistic model is employed by considering time as the 2nd level within Bayesian framework so as to make the comparison equally. Therefore, DIC is used to compare the models abovementioned:*

$\text{DIC}=D(\bar{\theta})+2p_{D}=\bar{D}+p_{D}$	*(7)*

*where* $D(\bar{\theta})$*is the deviance evaluated at* $\bar{\theta}$*, the posterior mean of the parameter of interest,* $p_{D}$ *is the effective number of parameter in the model, and* $\bar{D}$*is the posterior mean of the deviance statistic*$D(\bar{\theta})$*. The lower the DIC, the better the model fits. Generally speaking, differences in DIC of more than 10 definitely rule out the model with the higher DIC; differences between 5 and 10 are considered substantial, while the difference less than 5 indicates that the models are not statistically different from each other.”*

6. It seems that only three variables entered the final model. It would be great if the authors could conduct a comparison study among the proposed ordered logistic model, and some other state-of-art models, such as multinomial logit model, mixed logit model, etc.

**Response:** Followed the reviewer’s suggestion, several models have been tried, but in order to keep the ordinal feature, Bayesian multilevel ordered logistic model is employed by considering time as the 2nd level. The results are shown in Table 2.

**Table** 2 Parameter estimates for the proposed models

| **Variable** | **Bayesian multilevel ordered logistic** | | | | **Bayesian ordered logistic** | | | |
| --- | --- | --- | --- | --- | --- | --- | --- | --- |
|  | Mean | Std. Dev. | MCSE | 95% BCI | Mean | Std. Dev. | MCSE | 95% BCI |
| Crash location | -0.467* | 0,201 | 0.013 | (-0.863,-0.067) | -0.409^*^ | 0.207 | 0.009 | (-0.831,-0.021) |
| Time | 0.896* | 0.365 | 0.026 | (0.205, 1.637) | 1.000^*^ | 0.372 | 0.025 | (0.224,1.724) |
| Crash season | 0.501* | 0.211 | 0.025 | (0.102,0.910) | 0.554^*^ | 0.209 | 0.009 | (0.160,0.964) |
| Cut1 | -2.127 | 0.798 |  |  | -1.785 | 0.773 |  |  |
| Cut2 | 1.184 | 0.765 |  |  | 1.494 | 0.732 |  |  |
| Sigma^2^ | 0.277 | 0.460 |  |  |  |  |  |  |
| **Goodness-of-fit** | | | | | | | | |
| **No. of observations** | | 133 | | | 133 | | | |
| **DIC** | | 223.312 | | | 222.297 | | | |
| **Log marginal likelihood** | | -123.916 | | | -124.731 | | | |

7. There are a lot of typos and grammar errors. I would highly recommend the authors to get editing help from someone with full professional proficiency in English. For example, P1 ln45, “fatality” shall be “fatalities”; P1, ln 46, P2, ln 2 “thus” is not a conjunction. Please use “and thus”; P2, ln 13 “tha”; P5 ln 13, “thresholds” shall be “threshold”; P6 ln33, “inury”

**Response:** The authors have asked English professionals for help and improvement, and revised all the typos and errors. Hope the paper is well completed as required.
