# Supplementary material for: Investigation of injury severity in urban expressway crashes: A case study from Beijing
Source: PLoS One. 2020 Jan 13;15(1):e0227869. doi: 10.1371/journal.pone.0227869 (PMC6957292; doi:10.1371/journal.pone.0227869)
Supplement: Attachment — Submitted filename: Response to Reviewers Comments-R2.doc [file pone.0227869.s002.doc]

# Responses to Reviewers’ Comments

Dear Editor,

# Thanks for the suggestions of our manuscript entitled with “*Investigation of injury severity in urban expressway crashes: A case study from Beijing*”. The revisions are as follows according to the reviewers’ comments and are highlighted with red color in the paper to reveal the improvement.

We greatly appreciate your time and efforts to accept our manuscript for publication. Please contact me freely if any problems come up.

Best regards

Xuecai Xu

**Reviewer #1**: My previous concerns have been well addressed. And I have no further comments or suggestions. The paper can be published as its current format.

**Response:** Thanks for the reviewer’s approval.

**Reviewer #2:** Thanks for the authors' carefully addressing my comments in last round-review. However, there do have several problems in the revised manuscript, as follows.
(1) Page 5, Eq (2), the constraint on z value shall be a combination of one open interal and one closed interval;

**Response:** Thanks for the reminding. Equation (2) has been revised.

(2) Page 5, ln16, the application of multilevel ordered logistic model shall be better motivated and described. In the result analysis, the multilevel model performs almost the same as the ordered model.

**Response:** Followed the suggestion, the motivation of multilevel ordered logistic model has been added.

*“However, the injury severity levels may vary across spatial location, e.g. severity levels may be higher at some expressways while lower at others. In such cases, the extent of the effect of severity levels may be different. At this point, in statistical terms, there exists within-individual homogeneity and between-individual heterogeneity in the hierarchically structured data, and multilevel modeling approach provides an appropriate analytical framework to deal with the spatial issue. In this study, the basic cross-sectional ordered logistic model as the first level, and then the model development expands the basic model by adding the panel data to explain the between-expressway heterogeneity, which specifies the random intercept sigma2at the expressway level.”*

(3) In Table 2, where is the Coef and Std Err as described in the note information.

**Response:** Thanks for the reminding. The Coef. and Std. Err. are left from previous model, which has been removed.
